# Erratum

**DOI:** 10.1155/DTE.3.193

**Published:** 1997


					Diagnostic and Therapeutic Endoscopy, 1997, Vol. 3, p. 193
Reprints available directly from the publisher
Photocopying permitted by license only
(C) 1997 OPA (Overseas Publishers Association)
Amsterdam B.V. Published in The Netherlands
by Harwood Academic Publishers
Printed in Singapore
ERRATUM
In the paper entitled Synchronous Quadruple Lung Cancer Treated Curatively by Photodynamic Therapy" by
Makoto Saito et al., published in Vol 3. No.2, pp. 115-119. Figure 2B had been published incorrectly. The correct
figure is presented below.
FIGURE 2 Histological specimens stained by haematoxylin-eosin from the bifurcations between right B and B2 (a, x200), between right
B4 and B5 (b, x 100), between left B + 2 and B3 (c, x l00), and cytological specimens stained by Papanicolaou's method from the bifurca-
tion between left B6b and B6c (d, x400).
193

				

